# Leadership effectiveness through coaching: Authentic and change-oriented leadership

**DOI:** 10.1371/journal.pone.0294953

**Published:** 2023-12-06

**Authors:** Peter R. Halliwell, Rebecca J. Mitchell, Brendan Boyle

**Affiliations:** 1 Newcastle Business School, University of Newcastle, Callaghan, NSW, Australia; 2 Health & Wellbeing Research Unit, Macquarie Business School, Macquarie University, Sydney, NSW, Australia; University of Central Punjab, MALAYSIA

## Abstract

There has been an increasing shift towards individually owned leader development programs within organizations. Whilst leadership coaching is one of these and is gaining in popularity, the mechanisms of its effect remain poorly understood. We develop and investigate a model in which leadership coaching enhances leader effectiveness through coaching’s positive effect on authentic and change-oriented leadership behaviours as well as self-efficacy. To assess the model, multi-source data were collected for organizational leaders (N = 70) pre- and post-coaching. To investigate mechanisms of coaching’s effect, relations between latent change scores were assessed in structural equation modelling using partial least squares indicating that after accounting for base-line scores, coaching-related increases in authentic leadership behaviour has the largest total effect on leadership effectiveness.

## Introduction

There is significant evidence demonstrating the importance of leadership in organizations [1] however, authors such as DeRue & Myers [2] contend that research in leader development is lagging and has yet to produce the insights required to address a mounting leadership talent crisis. Further, changes made from leader development programs are often short-lived [3], and that many programs lack the impact relative to their costs. One intervention increasing in popularity, is leadership coaching [4], which seeks to address the short-lived nature of other development approaches by creating sustained behaviour change that results in lasting increases in leader effectiveness [5]. However, whilst leadership coaching is deemed to be amongst the most effective leader development practices [6] with studies indicating coaching is effective in achieving affective, skills-based and individual-level outcomes [7, 8], scholars posit that the practice of coaching e is ahead of its scientific understanding [9].

Research evidence for how and why leadership coaching increases leader effectiveness provides the basis that successful practice requires [10] and yet there are relatively few rigorous studies that provide longitudinal evidence for the link between leadership coaching and targeted outcomes and even fewer that shed light on the mechanisms through which leadership coaching renders its effect [9, 11–13]. Consequently, there is a recognised need for such research to shift coaching from a practice-driven discipline towards a field that is underpinned by scientifically credible and theoretically driven research [14].

**Fig 1:**
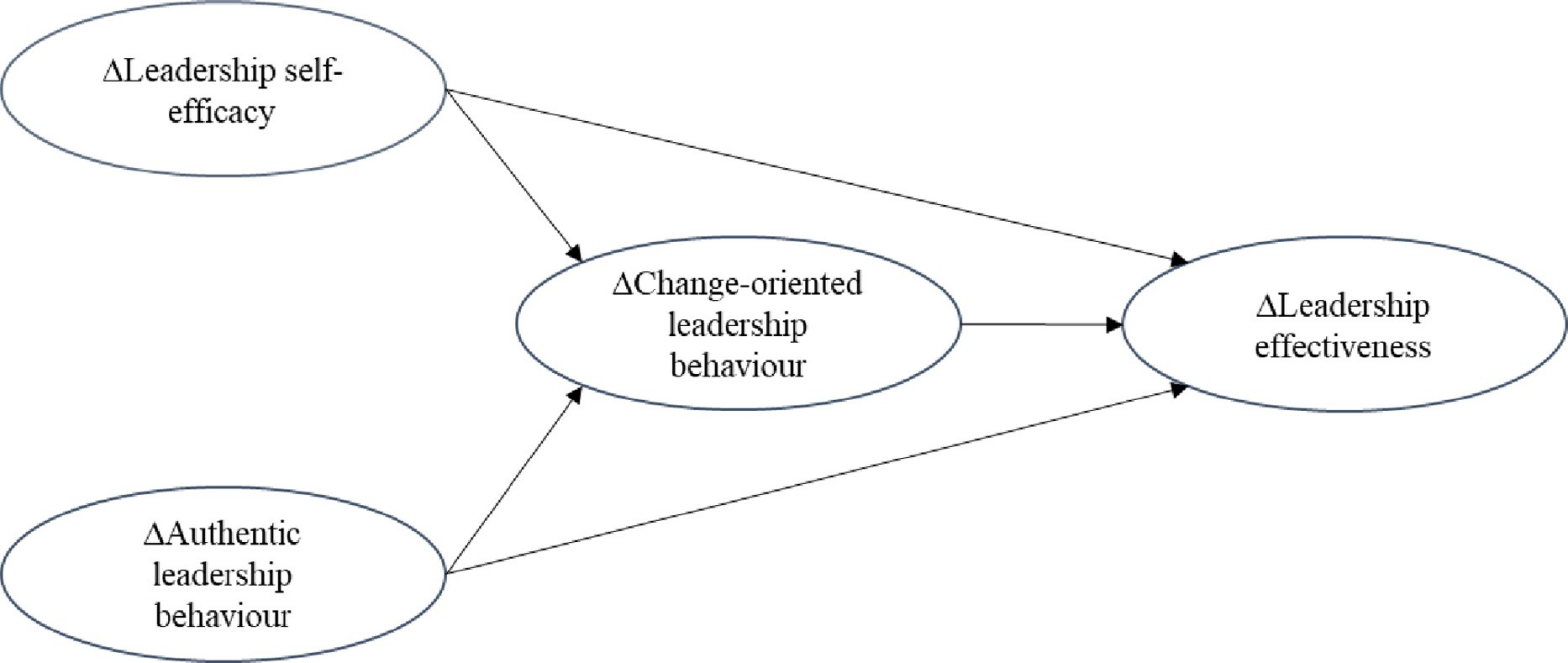
Conceptual model.

We respond to this research gap and calls for studies to empirically validate coaching interventions through multi-source longitudinal data as well as to investigate the mechanisms of its effect on leadership [12, 14, 15]. We respond to calls for studies to empirically validate coaching interventions through multi-source longitudinal data [16] and to investigate the mechanisms of its effect on leadership [15]. We undertake a quantitative pretest-posttest study that utilizes data sourced from coaches, coachees, subordinates and supervisors. We develop and investigate the impact of coaching-related increases in leadership self-efficacy and authentic leadership behaviour on increases in change-oriented leadership behaviour and leader effectiveness. This conceptual model is depicted in Fig 1. Undertaking this research allows us to advance our understanding of how and why leadership coaching builds leader effectiveness and provides specific insight into the impact of coaching on leader effectiveness through authentic and change-oriented leadership [17, 18].

### Leader development and coaching

Whilst multiple definitions have been used to define leadership coaching, common elements exist, such as a one-on-one relationship, raising self-awareness, performance, learning and development, and behavioural change [10]. Similarly, Jones et al. [7] identified core features of coaching, that is, a supporting relationship, setting personal development objectives, achievement of these objectives through focusing on inter- and intra-personal issues, and helping the coachee develop and be more effective by providing the coachee with the tools, skills and opportunities they need [8]. Through the application of these common elements, the coach becomes a supportive and competent thinking partner for the coachee, who through their interpersonal skills and utilisation of adulting learning techniques such as reflective questioning, encourages self-reflection and questioning of assumptions to enable the coachee to make sense of the information available and apply their personal learning to make sustainable positive change [19].

Accordingly, and drawing on reviews of the coaching literature [8, 20], we define leadership coaching as a one-on-one personalized learning and developmental intervention for organizational leaders that is based on a coachee-coach relationship characterized by collaboration, reflection and goal-focused dialogue to initiate and maintain positive change in coachee attributes and behaviour that precipitate the accomplishment of professional outcomes, and argue that coaching-related increases in authentic leadership behaviour and leadership self-efficacy will explain the impact of coaching on change-oriented leadership behaviour and overall effectiveness.

### Coaching-related authentic leadership effects

Authentic leaders are defined as leaders who have a clear sense of purpose, who practice enduring values such as integrity, who lead with the heart, who establish lasting, stable relationships and demonstrate self-discipline [21]. Coaching is capable of influencing factors associated with authentic leadership behaviour such as self-awareness, self-discipline and self-confidence [10, 22–24], emotional competencies [22], and clarity of objectives and purpose [25–27]. These factors characterise authentic leaders recent research has evidenced a significant association between leadership coaching and increases in authentic leader behaviors [13].

Leadership research indicates that authentic leaders are more respected, trusted and highly regarded by others and less likely to resist change [28], and have followers that exhibit higher levels empowerment, job performance, job satisfaction and organizational commitment [29]. Further, Avolio & Gardner [30] argue that authentic leadership is the basis or root construct of positive leadership models such as transformational, charismatic, and servant leadership, which have been associated with leadership effectiveness [31, 32]. Therefore, we argue that coaching-related increases in authentic leadership behaviour are likely to be associated with increases in leader effectiveness.

**Hypothesis 1a:** Coaching-related increases in authentic leadership behaviour are positively associated with increased leadership effectiveness.

We also argue that as leaders’ authentic leadership behaviours are increased, such as behaving with a clearer sense of purpose, being more transparent and honest in their actions and motives, seeking and processing information with less bias [33], and being more self-disciplined, leaders are likely to exhibit more change-oriented leadership behaviour such as articulating a clear vision, inspiring others to innovate, encouraging diversity of views, and facilitating collective learnings. Change-oriented leadership behaviour is exemplified by leaders who monitor the environment, propose ideas for change, encourage innovative thinking and persevere to overcome obstacles [34].

The impact of coaching-related increases in authentic leader behaviors on change-oriented leadership is supported by reviewing components making up authentic leadership behaviour identified by Avolio, Gardner & Walumbwa [35, 36], where for example, increases in transparency when leading others, taking a more balanced perspective and leading with increased morality, are likely to lead to enhance change-oriented leadership behaviour. Further, as leaders increase ‘congruence’ between their actions and true beliefs and values as exemplified in authentic leadership [37, 38] through coaching [39], leaders are likely to more strongly defend their position and influence others, that is, display increased change-oriented leadership behaviour. Accordingly, we hypothesise:

**Hypothesis 1b:** Coaching-related increases in authentic leadership behaviour are positively associated with increased change-oriented leadership behaviour.

### Coaching-related self-efficacy effects

Self-efficacy is concerned with belief in one’s capability to successfully perform at a given level [40]. Leadership coaching includes expressing confidence in the coachee’s capabilities and progress [23, 41, 42], verbally recognising the coachee’s achievements during the intervention [43] and encouraging the coachee to focus on their strengths and develop solutions to help the coachee achieve their goals [44, 45]. This suggests that leadership coaching will increase leader self-efficacy and this effect has been supported in several recent studies [e.g., 46].

Supported by a growing body of research, there is substantial evidence that self-efficacy has a significant impact on behaviour [47], and influences the choices people make and the effort and persistence people put in [48]. For example, Bandura [40] found those with higher self-efficacy tended to perceive difficult tasks as challenges that can be controlled and mastered, had higher quality analytical thinking and higher coping strategies. In the context of this study, we argue that coaching-related increases in a coachee’s efficacy to lead (leadership self-efficacy), are likely to be associated with increases in coachee’s efforts and persistence to lead more effectively, and more able to cope with challenging situations, leading to increased leadership effectiveness. Accordingly, we hypothesise the following:

**Hypothesis 2a:** Coaching-related increases in leadership self-efficacy are positively associated with increased leadership effectiveness.

Further, we argue that coaching-related increases in leader self-efficacy will mean that leaders are more confident at facilitating diverse views [47], more likely to challenge, motivate and convince others [49], and make increased efforts to understanding others’ states and needs [50], which, we propose, will lead to enhanced change-oriented leadership. Further, self-efficacy is associated with heightened confidence in one’s capability to understand and articulate the reasons behind decisions and will therefore be associated with a stronger defence of their position whilst encouraging diversity of views and consideration of others, and are likely to be more effective, and exhibit increased change-oriented leadership behaviour [51]. Accordingly, we hypothesise:

**Hypothesis 2b:** Coaching-related increases in leadership self-efficacy are positively associated with increased change-oriented leadership behaviour.

### Coaching related change-oriented leadership effects

Leaders with increased change-oriented leadership behaviour are more likely to encourage innovative thinking and persevere to overcome obstacles [34]. These are likely to result in increases in leadership effectiveness and organizational performance as change-oriented leaders are more likely to promote and encourage innovative strategies to build competitive advantage and facilitate change processes to enable adaption to environmental changes [52]. Further, change-oriented leadership behaviours are exemplified in charismatic and transformational leadership styles [53] which have been shown to be positively associated with leadership effectiveness [e.g., 31]. In addition, change-oriented leadership behaviours such as assessing the environment and challenging traditional thinking [17, 51, 54] leading to increased leadership effectiveness through improved competitive advantage, environmental adaptability and organisational performance [52]. Accordingly, we hypothesise:

**Hypothesis 3:** Coaching-related increases in change-oriented leadership behaviour are positively associated with increases in leadership effectiveness.

## Materials and method

### Participants and procedure

To test our hypotheses, a non-experimental within-subjects pretest-posttest design was adopted [55]. In total, 82 organizational leaders (coachees) predominately from Australia, participated in the study with data collected over 2017–2018. All participants gave informed implied consent as set out in the participant information statement they were provided with under the ethics approval of University of Newcastle Australia, protocol H-2015-0126. Twelve (14.6%) coachees did not have complete pre-post coaching data sets and were dropped from the analysis. For the remaining 70 coachees, almost half (49%) were men and 51% were women. Nearly three-quarters of our participants (73%) had a Bachelor or higher degree. They were, on average 44.2 years old (between 28 to 66 years) and had considerable leadership experience (13.2 years on average; an average of 13.2; SD = 10.0). Just over one-fifth (21%) classified themselves as front line supervisors, 59% as middle managers and 20% as senior or executive managers. Based on the ‘ten times rule’ for PLS-SEM [56] which advocates a minimum sample size of ten times the maximum number of independent variables in the outer model and inner model, a minimum of 70 participants is required. Whilst the study’s sample of 70 is the lower bound recommended, data for the dependent variables comprise of multi-rater assessments and latent change scores derived from pre- and post-coaching scores with high test-retest reliability, therefore considered to provide adequate estimate stability and statistical power.

To reduce the likelihood of common method bias which may threaten the validity of the study’s conclusions, several design elements were included as recommended by Podsakoff, et al. [57]. Our data were collected from different sources, i.e., from coaches (N = 8), coachees (N = 70), their supervisors (N = 59) and subordinates (N = 175), via online questionnaires at Time 1 (pre-coaching) and Time 2 (post-coaching) with scale format and anchors differing for each construct. To reduce socially desirable responses, respondents were asked to answer as honestly as possible, and were advised that their anonymity and confidentiality would be assured. To reduce response bias from experimenter demands such as coachees altering their behaviours based on cues on the study’s objectives and/or consciously or unconsciously attempting to productively participate and do well in the study [58], two of the constructs were measured by others and data were collected for four additional constructs which were not assessed in this study.

Each coachee received an average of six one-on-one coaching sessions which lasted between 60–90 minutes. Coaching was received over an average of four months to achieve developmental (46%), skill development (30%), performance improvement (11%) or other (13%) goals. Coaching sessions were delivered by full-time and accredited leadership coaches having on average 14 years coaching experience, were external to the coachees’ organizations, and primarily identified their coaching as being informed by solutions-focussed and adult learning coaching frameworks [see 59, 60].

### Measures

Following recommendations for social sciences research, a latent variable model was utilised [61]. The study’s four latent variables were operationalised using questionnaires predominately from prior leadership studies due their established reliability and validity.

Leadership self-efficacy was assessed with the Leader Efficacy Questionnaire (LEQ) developed by Hannah & Avolio [62]. The LEQ was completed by the coachees and consists of 22 items. Participants are asked to indicate their level of confidence ranging from 0 = not at all confident, 100 = totally confident. The LEQ measures three components of leadership self-efficacy, i.e., leader action self-efficacy (SEa), leader self-regulation efficacy (SEr) and leader means self-efficacy (SEm), which we consider to be distinct components and not interchangeable, therefore Leadership self-efficacy was operationalised as a reflective-formative second-order construct [63]. Higher order constructs also improve model parsimony and allow more detailed analyses of dimension-specific effects [64].

Authentic leadership behaviour was assessed with the Authentic Leadership Questionnaire (ALQ) developed by Avolio et al. [65]. The ALQ was completed by the coachee’s subordinates and utilises a 16 item 5-point Likert questionnaire (1 = not at all, 5 = frequently, if not always) measuring four components of authentic leadership behaviour, i.e., transparency (LAt), moral/ethical (LAm), balanced processing (LAb) and self-awareness (LAs). We consider these as distinct components and not interchangeable, therefore Authentic leadership behaviour was operationalised as a reflective-formative second-order construct.

Change-oriented leadership behaviour, utilising Yukl [51] and DeRue et al.’s [53] research into mega-categories of leadership behaviour, change-oriented leadership behaviour was assessed with four reflective items representing change-oriented leadership behaviour from Yukl’s [51] 15 item taxonomy of positive leadership behaviour (i.e. advocating change, articulating an inspiring vision, encouraging innovation, and encouraging collective learning). Each item utilised a 5-point Likert scale (1 = not at all, 5 = to a very great extent) with data provided by the coachee’s subordinates as they were most likely to observe the coachee’s behaviours due to their frequent work interactions with their supervisor.

Leadership effectiveness was assessed using the approach adopted by Hooijberg & Lane [66, 67] where ‘perceived’ leadership effectiveness is measured rather than attempting to assess performance of business units or departments for which the leader is responsible. Specifically, perceived leader effectiveness was assessed by the coachee’s subordinates and immediate supervisor with five reflective items developed by Hooijberg & Choi [68] and Yukl [69] measuring overall leadership effectiveness, the extent they met performance standards, how they performed as a role model, how they compared to their peers, and their effectiveness relative to the most effective leader they have known. To reduce the potential for response bias [70] three different scale structures were used to capture leader effectiveness. Three of the five items utilised 6-point Likert scales (1 = very ineffective, 6 = very effective), one utilised a 5-point Likert scale (1 = well below average, 5 = well above average) and one a 10-point Likert scale (1 = the least effective leader I have known, 10 = the most effective leader I have known).

### Control variables

Several variables have been found to influence coaching’s effects on a leader’s development, such as developmental support [71], coaching medium [72], number of coaching sessions [73], coaching approach [74] and coachee demographics [75]. Whilst data for these variables were collected, to ensure sufficient statistical power for the analyses, only four control variables exhibiting the largest variability were included, i.e., coachee age (range 28–66), coaching sessions (range 3–12), coaching duration (range 1.3–12), and organizational support (range ‘very poor’ to ‘very good’). Coachee age was provided by the coachee at Time 1, number of coaching sessions was provided by the coach at Time 2, coaching duration was calculated as the difference between the dates of the pre- and post-coaching surveys measured in months, and organizational support was assessed by coachees at Time 2 using a single 5-point Likert scale item (1 = Very poor, 5 = Very good).

### Analytical method

As the level of analysis was at coachee level, to ensure alignment of theory, measurement and analyses [76], aggregation of rater scores was required for each coachee. To ensure adequate within-group agreement prior to aggregation, r_wg(j)_ indices were calculated using a cut-off criterion of 0.70 [77]. Mean r_wg(j)_ values were satisfactory for change-oriented leadership behaviour which was scored by the coachee’s direct reports (r_wg(j)_ pre = 0.81, post *=* 0.83) and similarly for leadership effectiveness which was scored by the coachee’s direct reports and managers (r_wg(j)_ pre = 0.87, post *=* 0.90). To assess test-retest reliability, i.e. intra-rater reliability was required [78]. This was assessed through intraclass correlations coefficients (ICC(3,k); [79]), and calculated using two-way mixed effects analysis of variance. Results ranged from 0.831 for change-oriented leadership behaviour to 0.932 for authentic leadership behaviour suggesting satisfactory test-retest reliability [79]. Further, to address reliability concerns associated with using raw change score in assessing structural models such as regression to the mean effects [80], latent change scores (LCS: [81]) with multiple indicators were used to test the hypothesised model. LCS represent the part of the post-coaching score that is not predictable from the pre-coaching score and were calculated by regressing the post-coaching scores onto the baseline pre-coaching scores and subtracting the predicted value from the observed post-coaching score [82].

To assess relations between constructs in the conceptual model, LCS and structural equation modelling (SEM) using partial least squares (PLS SEM; [83, 84]) were assessed with SmartPLS 3.2.8 [85]. Due to the model complexity, structural equation modelling was determined to provide improved model estimates relative to first generation statistical techniques such as hierarchical regression modelling. Specifically, the variance-based SEM technique partial least squares structural equation modelling (PLS SEM) rather than covariance-based structural equation modelling (CB SEM) was utilised due to the predictive rather than confirmation nature of the study. Further, PLS SEM is commonly used in the HRM literature [86] where the research aim is predictive application and theory building, and due to its capacity to assess theoretical hierarchical constructs and relationship between these simultaneously [56]. For the higher-order constructs, the repeated indicator approach using mode A of measurement was used as it is considered conceptually superior to the two-stage approach when the higher-order constructs are in an exogenous position [87].

As data for authentic and change-oriented leadership behaviours were from a common source, i.e., the coachee’s subordinates, and data were collected at the same time using the same questionnaire, two post-hoc tests were performed to assess for common method bias. First, the Harman’s single-factor test [88, 89] which indicates that common method bias may exist when a single factor emerges amongst the first-order reflectively measured factors, produced 8 distinct factors, the largest of which accounted for 26.19% of the variance of the model. Second, a full collinearity test of the first-order constructs was undertaken to check whether all inner VIFs were less than 3.3 [90] and that there were no correlations among the formative constructs greater than 0.9 [91]. The highest VIF value was 1.68 and largest correlation was 0.621 (LAb and LAt). These post-hoc tests indicate the data did not suffer from common method bias.

## Results

### Measurement model

We assessed reflective constructs (including the reflective first-order dimensions of the high-order constructs) for their reliability, consistency and validity, and formative constructs for their collinearity, and the significance and relevance of their indicator weights. Indicators with low loadings were removed leaving 10 of 16 items measuring first-order dimensions of authentic leadership behaviour, four of five items measuring change-oriented leadership behaviour, four of five items measuring leadership effectiveness and 19 of 22 items measuring first-order dimensions of leadership self-efficacy. Scale reliability (internal consistency) was assessed using Cronbach’s alpha and Joreskog’s [92] composite reliability *p*_c_. Cronbach’s alpha values were higher than 0.6 and composite reliability greater than 0.70 but less than 0.95, indicating acceptable internal consistency [93]. Convergent validity was assessed by an examination of the average variance extracted (AVE) and calculated as the mean of the squared loadings for each construct’s indicators. All AVE values were satisfactory, i.e., greater than 0.50 indicating that on average, the constructs explained over 50% of the variance of their items [56, 94]. Discriminant validity was assessed utilising the Fornell and Larcker [95] and heterotrait-heteromethod correlations criteria (HTMT; [96]) to ensure reflective constructs have the strongest relationships with their own indicators [97]. The highest HTMT value of 0.848 (LAt—LAm) is less than 0.90 and based on bootstrapping 5,000 samples was significantly less than 1 indicating the model’s reflective constructs are sufficiently dissimilar [96]. Further, an examination of cross loadings whereby an indicator should exhibit a higher loading on its own construct than any other construct [93], suggests adequate discriminant validity exists for the reflective constructs.

To assess the level of collinearity among the formative indicators, variance inflation factors (VIF) were calculated. VIF values ranged from 1.502 to 1.778 for the 2^nd^-order formative indicators for authentic leadership behaviour and 1.211 to 2.229 for the 2^nd^-order formative indicators for leadership self-efficacy. These are well below the cut-off value of 5 indicating collinearity is not present [56]. Next, the significance and relevance of the formative indicators were assessed. Indicator weights ranged from 0.316 to 0.338 for the 2^nd^-order formative indicators for authentic leadership behaviour and similarly 0.172 to 0.509 for leadership self-efficacy. The bootstrap procedure [98] with 5,000 samples was used to calculate *p*-values for the indicator weights. All were statically significant (*p*<0.001) confirming formative indicator significance and relevance.

### Hypothesis testing

Table 1 presents means and standard deviations of constructs at Time 1 & 2, results of paired t-tests and Cohen’s effect sizes. Results indicate means for all constructs increased from Time 1 to Time 2 and were statistically significant at the alpha = 0.05 level. Effect sizes for the mean differences of authentic leadership behaviour, change-oriented leadership behaviour and leadership effectiveness are considered small to moderate in behavioural research [99], however for leadership self-efficacy are considered large.

**Table 1:** Means, standard deviations, paired t-tests and effect sizes.

	T1		T2		Paired *t*-test & Effect size		
	M	SD	M	SD	*t*-value	*p* value	Cohen’s D
Authentic leadership behaviour	62.4	11.9	63.7	11.3	2.42	0.017	0.18
Leadership self-efficacy	176.7	22.8	193.6	22.5	8.38	0.000	1.01
Change-oriented leadership behaviour	15.1	3.3	15.4	3.4	2.33	0.021	0.15
Leadership effectiveness	26.3	4.8	26.9	4.5	3.28	0.001	0.22

PLS SEM was employed to investigate the hypotheses. First, the structural model was assessed for potential collinearity between the predictor constructs, as if present may bias the estimation of path coefficients [93]. This was assessed through VIF values which ranged from 1.052–1.274 indicating collinearity is not present as these values are less than 5 [100, 101]. Next, to assess model performance, an evaluation of in-sample prediction quality was assessed by evaluating the endogenous constructs’ variance explained (R^2^), their bias-corrected bootstrapped confidence intervals, path effects sizes (f^2^), and the constructs’ cross-validated predictive relevance (Stone-Geisser’s Q² value; [102, 103]) utilising the sample re-use technique blindfolding [64]. As we seek to generalise the results beyond the sample, an evaluation of out-of-sample prediction was also undertaken utilising Shmueli et al.’s [104] PLS predict procedure. In this procedure, the smaller the difference between the predicted and the original values, the greater the Q^2^ value, and therefore the model’s predictive accuracy. Results are presented in Table 2 and indicate that the model has adequate in-sample and out-of-sample predictive relevance, although the variance explained (R^2^) and predictive relevance (Q^2^) for increased change-oriented leadership behaviour are considered weak, however results for increased leadership effectiveness are considered moderate to strong [105]. As PLS-SEM aims at maximizing the explained variance of the dependent variables, model quality criteria cannot indicate goodness-of-fit or a lack thereof in a CB-SEM sense, therefore model fit statistics are not presented [106, 107].

**Table 2:** Model performance (Q^2^ & R^2^).

	Q^2^ (Blindfold)	Q^2^ (PLSpredict)	R^2^	R^2^ (2.5% CI)	R^2^ (97.5% CI)
ΔChange-oriented leadership behaviour	0.100	0.001	0.218	0.045	0.331
ΔLeadership effectiveness	0.166	0.002	0.315	0.127	0.415

Notes: *Δ* = “changes in” represented by latent change scores; SmartPLSv3.2.8 settings: Maximum iterations = 300; Stop criteria = 7; Blindfold omissions distance = 8; PLSpredict repetitions/folds = 10/10; Significant = 0.05; Test type = 2 tailed

**Fig 2:**
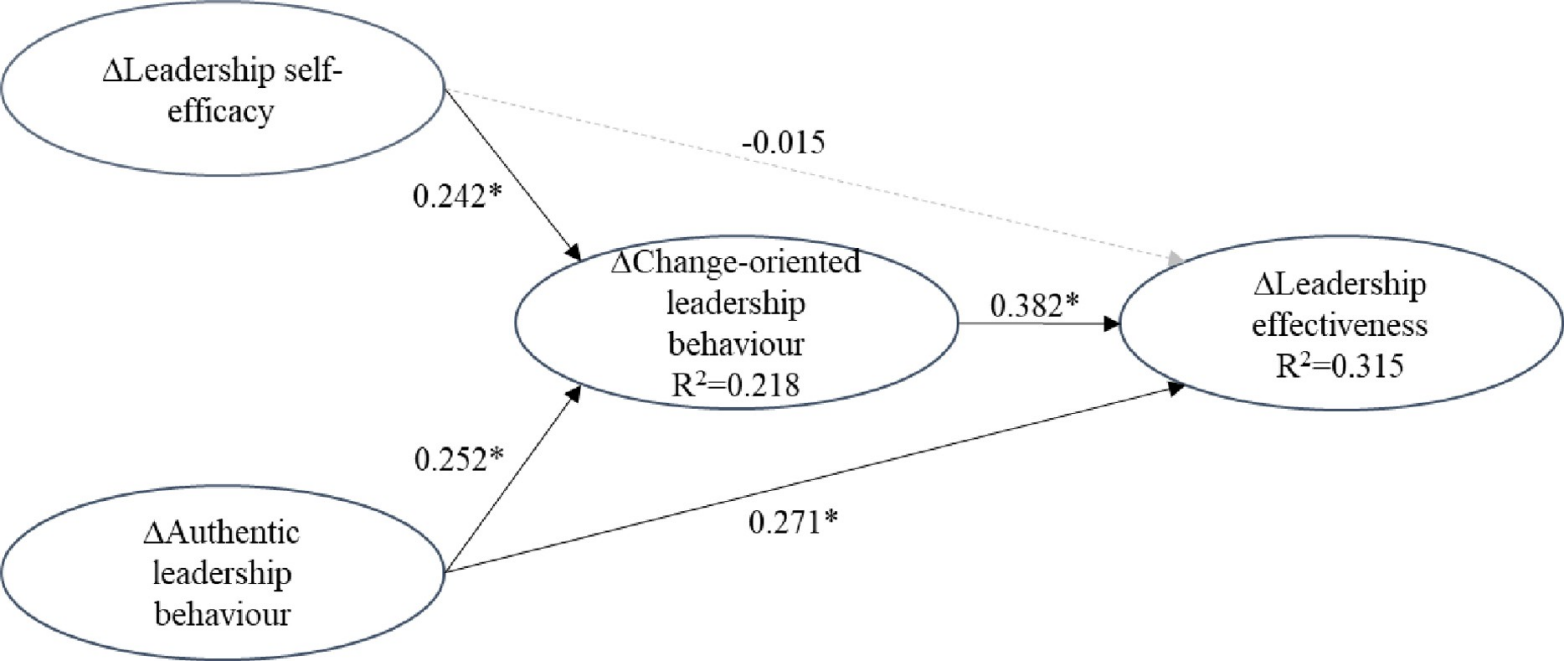
PLS SEM estimation results. Notes: Δ = = “Changes in” represented by latent change scores; Results on paths are standardised *β* weights; *p<0.05; Dashed lines represent non-significant paths.

Finally, the strength, sign and significance of the path coefficients were evaluated for the hypothesised relationships between the constructs. To assess direct effects, the non-parametric bootstrapping procedure was applied in SmartPLS (5,000 samples, no-sign change option, and two-tailed test), controlling for organizational support, number of coaching sessions, coaching duration and coachee age. Our PLS SEM analysis, reported in Table 3 and Fig 2 provides support for positive associations between coaching-related increases in authentic leadership behaviour and increases in leadership effectiveness (*β* = 0.271, *t =* 1.965, *p =* 0.049) and change-oriented leadership behaviour (*β* = 0.252, *t =* 2.019, *p =* 0.044) supporting hypothesis 1a and 1b. Support for hypotheses 2b was also found, indicating a positive relationship between coaching-related increases in leadership self-efficacy and increases in change-oriented leadership behaviour (*β* = 0.242, *t =* 2.004, *p =* 0.045), however no support was found for hypothesis 2b as a positive association between increased leadership self-efficacy and leadership effectiveness (*β* = -0.015, *t =* 0.888, *p =* 0.144). In support of our third hypothesis, evidence was found for a positive relationship between increased change-oriented leadership behaviour and increased leadership effectiveness (*β* = 0.382, *t =* 2.356, *p =* 0.019). Except for a positive association between organizational support and increased leadership effectiveness (*β* = .235, *t =* 2.456, *p =* 0.014), there was no evidence of significant associations between the control variables and increased change-oriented leadership behaviour or increased leadership effectiveness.

**Table 3:** Estimated path coefficients.

	without control variables			with control variables		
	β	*p* value	*t* value	β	*p* value	*t* value
ΔSE = >ΔLC	0.257	0.030	2.177	0.242	0.045	2.004
ΔSE = >ΔLeff	0.100	0.306	1.023	-0.015	0.885	0.144
ΔLA = >ΔLC	0.276	0.034	1.845	0.252	0.044	2.019
ΔLA = >ΔLeff	0.365	0.023	2.126	0.271	0.049	1.965
ΔLC = >ΔLeff	0.215	0.065	2.275	0.382	0.019	2.356
Age = >ΔLC				0.189	0.093	1.679
Age = >ΔLeff				-0.174	0.108	1.605
Duration = >ΔLC				0.195	0.169	1.374
Duration = >ΔLeff				-0.125	0.270	1.103
Sessions = >ΔLC				0.153	0.155	1.423
Sessions = >ΔLeff				0.167	0.110	1.600
Support = >ΔLC				-0.044	0.674	0.420
Support = >ΔLeff				0.235	0.014	2.456

Notes: *Δ* = “changes in” represented by latent change scores; SE = Leadership self-efficacy, LA = Authentic leadership behaviour, LC = Change-oriented leadership behaviour, Leff = Leadership effectiveness.

## Discussion

The objective of this study was to further empirical research on the impact of coaching on leadership. We developed and investigated a model in which leadership coaching changes in authentic leadership and self-efficacy enhanced change-oriented leadership and leadership effectiveness. Following an assessment of 70 organizational leaders pre- and post-coaching, we assessed relations between construct latent change scores. Specifically, following confirmation of the reliability and validity of the latent change score constructs and satisfactory predictive power of the structural model, PLS SEM results provide support for hypothesised effects. These results indicate, that after accounting for leaders’ baseline scores, coaching-related increases in leadership self-efficacy and authentic leadership behaviour are positively associated with increases in change-oriented leadership and increased authentic and change-oriented leadership behaviours are positively associated with increased leadership effectiveness. Increased authentic leadership behaviour was found to have the largest total effect on leadership effectiveness.

### Theoretical contribution

This study makes several important theoretical contributions. The main contribution of our findings stem from increased insight into the mechanisms through which coaching yields its effect on leadership. Responding to calls from scholars to investigate the mechanisms for coaching’s effect [e.g., 15], this study hypothesised a complex model of leadership effectiveness. Whilst support was found for posited direct effects, causation cannot be claimed due to the study’s non-experimental design. However, this study does provide some evidence supporting hypothesised mechanisms for coaching’s effect. We find evidence that changes in authentic leader behaviors influence the effectiveness of leaders as well as their capacity to adopt change-oriented leadership styles. While previous studies have linked coaching to increased authentic leader behaviors [46], we substantially extend this research by evidencing the significant impact of enhanced authentic leadership on overall effectiveness. Further, we contribute to the authentic leadership literature by supporting the role of leader authenticity in change-oriented leadership and provide insights into the mechanism through which leader coaching can enhance this important leadership style [17]. This contribution is particularly compelling given the positive impact of change-oriented leadership, particularly in dynamic contexts or those requiring learning and adaptation [54, 108].

Our finding that coaching-related self-efficacy increases change-oriented leadership makes a similarly useful contribution by adding to our understanding of how self-efficacy can increase the breadth of leadership styles available to recipients Though coaching-related self-efficacy has been evidenced previously [13], our findings provide a meaningful extension by highlighting the flow-on effects of this internal, self-regulatory change. Our findings lend support to coaching frameworks such as Leedham’s [25] pyramid model which suggests inner proximal benefits such as increased awareness and confidence precede outer distal benefits such as improved self-regulation and behaviour, leading ultimately to improved performance. This is particularly relevant in the context of Ladegard and Gjerde’s [109] assertion that leadership development could be accelerated if organizations focussed on the “interior processes and less on exterior and observable competencies” (p.14). Whilst there was no support for a positive association between increased leadership self-efficacy and leadership effectiveness, this may reflect insufficient temporal separation between pre- and post-assessments for changes in this cognition to be observed by others. This is consistent with Spence et al.’s [19] (2019) research which suggests that leadership coaching, given its reflective nature, coachees may require time to consolidate and apply learnings, and therefore may not be immediately translated into behaviour and effectiveness.

Further, responding to calls for more rigorous studies to be undertaken on leader intervention studies [110], and in particular, on leadership coaching where empirical support lags its practical application [75], we employed a pretest-posttest design utilising multi-source data to assess coaching effectiveness. Results add further support to research indicating that coaching may be an effective development intervention [111]. In addition, this study extends the coaching literature into the domains of authentic and change-oriented leadership behaviours by providing evidence that participation in coaching is associated with increases in these important leadership capabilities. Acknowledging Avolio & Gardner’s [30] work indicating leader development should be centred on authentic leadership behaviour, and challenges today’s leaders face in managing change [112], this is a particularly relevant extension of the literature.

### Practical implications

Whilst there are many leader development interventions being used across organizations such as job assignments, 360-assessments, coaching and formal leadership development programs [113], more research is needed to determine their effectiveness [114]. Further, concerns have been raised on the sustainability of changes made from formal leader development programs resulting in a trend towards leader-owned development within organizations. This study assists organizations wanting to develop their leaders using leader-owned development programs, by providing additional evidence for the effectiveness of leadership coaching. For example, noting the large effect sizes found in this study for leadership self-efficacy and its positive association with change-oriented leadership behaviour, coaching presents a potentially valuable intervention to assist leaders tasked with leading organizational change.

Whilst our study indicates coaching has a large effect on leadership self-efficacy, its smaller effect on authentic leadership behaviour are particularly interesting noting evidence found for positive associations between increased authentic leadership behaviour and increased change-oriented leadership behaviour and leadership effectiveness. This suggests that HRD practitioners and organizations wanting to increase the effectiveness and career development of their leaders, may find significant value in engaging leadership coaches, and that coaches tasked with improving a leader’s effectiveness such as through their ability to lead change, may find value in focussing on improving aspects making up authentic leadership behaviour such as self-regulated attention [115]. This may be particularly relevant noting concerns on organizational leadership reflected in scandals such as Enron’s collapse and VW’s diesel gate saga, and research indicating trust in leaders which has been associated with authentic leadership behaviour, may assist to reduce employee turnover and increase knowledge transfer within organizations [116]. In sum, coaching’s effects on authentic leadership may present wider and more significant benefits to organizations than those originally hypothesised.

### Limitations and directions for future research

As the study assessed coachees across multiple organizations to improve external validity, due to limitations on resources, control groups were not incorporated. Accordingly, causality cannot be claimed in this study and alternate explanations beyond the coaching intervention may have contributed to changes in observed between Time 1 and 2 such as experimenter demand effects [58]. Future studies would benefit by adopting randomised control group experimental designs to assess causality [117]. Future studies would also benefit from a third and longer time point to allow enough temporal separation for changes in cognition to be translated into effectiveness [118]. A third time point would also provide additional information on the shape and slope of development trajectories.

We also acknowledge that limited coaching controls and eligibility requirements were incorporated into the study. Whilst several control variables were incorporated to account for their potential effect, differences between coaches, coachees and the coaching provided, have the potential to affect coaching outcomes. Accordingly, future studies would benefit from the inclusion of additional controls and eligibility requirements, and from a larger sample size to control for and assess their potential effects. A larger sample size would also assist to test for unknown heterogeneity, and for multi-group analyses such as comparisons of effects between differing levels of leader seniority. Finally, whilst this paper investigates coaching’s effects on several important leadership constructs and the relations between changes observed, investigation of coaching’s effects on other leadership constructs is needed to continue building the literature on coaching and leader development.

Despite these limitations, our research provides significant insight into the flow-on effects of coaching-related increases in self-efficacy and authentic leadership. Such insights provide a sound base for future research and contribute to the growing evidence supporting effective leadership development coaching practice. More specifically, support for our modelled relationships allow a clearer picture of how coaching can increase change-oriented leadership capability and broader leader effectiveness.
